# A Canadian Perspective

**DOI:** 10.1371/journal.pmed.0020083

**Published:** 2005-03-29

**Authors:** James E Till

**Affiliations:** University of TorontoToronto, OntarioCanada

Erick H. Turner [1] notes that “ClinicalTrials.gov, a registry authorized by the Food and Drug Modernization Act of 1997, appears not to be comprehensive.” While we await the creation of clinical trials registry and results databases that are truly comprehensive, innovative efforts to provide convenient access to credible information about known existing clinical trials need to continue. A Canadian example is provided by OntarioCancerTrials.ca, a consumer-oriented site developed by the Ontario Cancer Research Network (OCRN), with funding from the Ontario government.
